# Efficiency, Microbial Communities, and Nitrogen Metabolism in Denitrification Biological Filter: Insights into Varied Pore Ceramsite Media

**DOI:** 10.3390/microorganisms13061187

**Published:** 2025-05-23

**Authors:** Jiajun Song, Na Yu, Cui Zhao, Yufeng Lv, Jifu Yang

**Affiliations:** State Key Laboratory of Simulation and Regulation of Water Cycle in River Basin, Department of Irrigation and Drainage, China Institute of Water Resources and Hydropower Research, Beijing 100048, China; song110120@163.com (J.S.); yuna1@lcu.edu.cn (N.Y.); zhaocui@iwhr.com (C.Z.)

**Keywords:** pore structure, denitrification biological filters, extracellular enzyme polymers, microbial communities, nitrogen metabolism functions

## Abstract

This study presented an investigation into the role of ceramsite pore structures in optimizing DNBFs for nitrate-contaminated water treatment. Through systematic comparison of three ceramsite media (CE1, CE2, CE3) with distinct pore structures, we elucidated the microbial mechanisms underlying nitrate removal efficiency by analyzing denitrification performance, biomass accumulation, EPS, microbial community structure, and nitrogen metabolic function. Results demonstrated that the CE2 medium, characterized by an effective porosity (pore size > 0.5 μm) of 55.8% and an optimal porosity (pore size 0.5–25 μm) percentage of 83.1%, achieved superior nitrate removal efficiency (87.8%) with an R_vd_ of 0.82 kg TN/(m^3^·d) at HRT = 1.5 h, outperforming CE1 (0.74 kg TN/(m^3^·d)) and CE3 (0.68 kg TN/(m^3^·d)). Enhanced performance was mechanistically linked to CE2’s higher biomass accumulation (8.5 vs. 7.8 mg/m^2^ in CE1 and 6.9 mg/m^2^ in CE3) and greater EPS production (48.5 vs. 44.7 in CE1 and 35.4 mg/g in CE3), which facilitated biofilm resilience under hydraulic stress. Microbial analysis revealed CE2’s unique enrichment of a higher relative abundance of *Proteobacteria* (90.1% vs. 67.2% in CE1 and 47.4% in CE3) and denitrifying taxa (unclassified_f_Comamonadaceae: 42.7%, unclassified_f_Enterobacteriaceae: 35.3%). PICRUST2 showed 1.2- and 1.4-fold higher abundance of denitrification genes (*narGHI*, *nosZ*) compared to CE1 and CE3, respectively. These findings establish that optimizing ceramsite pore structure, particularly increasing the optimal porosity ratio (pore size 0.5–25 μm) can enhance denitrification efficiency, offering a scalable strategy for cost-effective groundwater remediation. This work provides actionable criteria for designing high-performance DNBFs, with immediate relevance to industrial and municipal wastewater treatment systems facing stringent nitrate discharge limits.

## 1. Introduction

Nitrate contamination, resulting from the oxidation of nitrogenous waste, has emerged as a significant water pollution issue, exacerbated by the indiscriminate discharge of industrial and domestic wastewater and the overuse of agricultural fertilizers [1]. Groundwater nitrate pollution has reached catastrophic levels in 30 regions across Africa, 20 in Asia, and nine in Europe [2]. High nitrate levels contribute to water body eutrophication and pose health risks, including haemoglobinopathies, thyroxine disorders, and potentially cancer, from long-term exposure [3]. To address nitrate pollution in groundwater, several removal strategies have been developed, including physical, chemical, and biological methods. Physical methods, such as ion exchange and reverse osmosis, transfer or concentrate nitrates but are costly, do not ensure complete removal, and carry a risk of secondary pollution [4]. Chemical methods, which use sulfate, iron powder, and thiosulfate to reduce nitrates to nitrogen, are quick but have a low conversion rate and also pose pollution risks [5]. In contrast, biological methods are gaining prominence for their environmental benefits and efficiency. They utilize organic carbon sources, like glucose and ethanol, or inorganic substrates, such as hydrogen and sulfur, to convert nitrates into nitrogen gas without harming the environment [6]. Denitrification biofilm filters, a key biological treatment technology, are widely applied in the advanced treatment of nitrate-rich waters. They offer advantages such as a small footprint, ease of operation, minimal suspension production, robust shock resistance, and high volumetric loading capacity [7].

The media in a denitrifying biofilm filter, serving as the microbial attachment substrate, are crucial for water treatment efficacy, with its physical properties—such as shape, size, surface area, and roughness—playing significant roles [8]. Spherical media like quartz sand [9], zeolite [10], gravel [11], and ceramsite granules [8] are ideal for biofilm formation due to their superior hydraulics, high attachment rates, and resistance to clogging. However, quartz sand, zeolite, and gravel have low porosity, hindering microbial attachment. It is necessary to increase the effective volume of filter to achieve high efficiency, which increases investment and management complexity. Ceramsite, a common filter medium, offers advantages of availability, low cost, high porosity and stable chemical properties [12]. Research has shown that ceramsite with particle sizes of 4–12 mm is particularly effective for denitrification [8]. While specific surface area is a key parameter for media attachment, it does not fully capture its impact on biofilm formation, as only a portion of the total surface area is accessible to microorganisms [13]. The growth of microorganisms is mainly influenced by media porosity and pore size, with the surface pore structure affecting biomass and extracellular polymeric substances (EPS) [14,15]. High porosity medium facilitates biomass accumulation, which in turn enhances denitrification efficiency [16]. The appropriate pore size allows for bacterial entry while avoiding excessive reduction of surface area for bacteria colonization. The optimal biomass accumulation is achieved with pore sizes from one to five times the microorganism size [17,18]. Given that the typical size of bacteria is between 0.5 and 5 mm [19,20], it can be inferred that pore sizes larger than 0.5 mm are capable of colonization by bacteria. Pore sizes ranging from 0.5 to 25 mm are considered optimal. In conclusion, ceramsite with high porosity and an appropriate pore size distribution provides more attachment sites for microorganisms, facilitating the colonization of microbial communities and improving nitrate removal efficiency. Zhen et al. employed porous ceramsite as a filtration medium to develop an innovative constructed wetland system, demonstrating superior water purification performance. The system achieved significantly enhanced removal efficiencies of 81.01% for COD and 90.25% for NH_4_^+^-N, outperforming conventional gravel-based wetlands by a substantial margin [21]. In a separate study, Yuan et al. conducted a 385-day experiment utilizing biofilm filters packed with ceramsite media for domestic wastewater treatment. Their results revealed that TN removal efficiency reached 80–86% under continuous aeration conditions [22]. Dong et al. investigated NH_4_^+^-N removal in an aerobic biofilter using ceramsite with a porosity of 49.8% as the filter material. Following biofilm maturation, the system attained a maximum NH_4_^+^-N removal rate of 85% [23]. Similarly, Ou et al. explored NH_4_^+^-N elimination in an aerobic biofilter employing ceramsite with a dominant pore size of 2 μm and a porosity of 48.57%. Over 231 days of continuous operation, the ceramsite-based filter maintained an average NH_4_^+^-N removal efficiency exceeding 90%, significantly surpassing the performance of quartz sand as a comparative filter medium [24]. In a focused investigation on denitrification processes, Liu et al. examined the nitrate nitrogen removal efficiency of ceramsite-packed filters under varying HRTs. Their findings demonstrated a NO_3_^−^-N removal rate of 79.9% at HRT = 0.5 h, with Acinetobacter and Methylophilaceae identified as the dominant microbial communities within the system [25]. Many scholars have studied ceramsite as filter media, but most research has focused on its application in aerobic filter environments. Additionally, existing studies provide insufficient descriptions of ceramsite’s pore characteristics, particularly regarding how its pore structure impacts microbial communities. Many scholars have studied ceramsite as a filter material, but most research has focused on its application in aerobic filter environments. Additionally, existing studies provide insufficient descriptions of ceramsite’s pore characteristics, particularly regarding how its pore structure impacts microbial communities. In particular, the relationship between pore structure of ceramides and microbial community dynamics has not been fully explored under different hydraulic regimes and elevated nitrate concentrations. Further research into the effects of ceramsite pore structure on denitrification filter efficiency and microbial communities is essential. This will not only optimize the design and operation of denitrification filters but also provide a theoretical basis for understanding the interactions between microorganisms and their environment.

In summary, while previous studies have explored the application of ceramsite in aerobic filter environments, limited research has focused on its role in denitrification systems, particularly regarding how pore structure impacts microbial communities and nitrogen metabolism. This study addresses this gap by investigating three distinct ceramsite media (CE1, CE2, and CE3) in denitrification biological filters (DNBFs). Our research question is: What is the role of ceramsite pore structure in optimizing denitrification efficiency in biofilm filters, and how do variations in pore characteristics influence microbial community dynamics and nitrogen metabolism functions? We hypothesize that optimizing the pore structure of ceramsite (particularly increasing the effective porosity and optimal pore size ratio) can enhance denitrification efficiency by promoting microbial attachment, biofilm formation, and the abundance of denitrification-related functional genes. The objectives of this study are: (1) to analyze the surface morphology and pore structure characteristics of ceramsite media before and after biofilm formation, (2) to evaluate the impact of pore structure variations on denitrification performance under different HRTs, (3) to explore the effects of pore structure on biomass accumulation and EPS composition, and (4) to characterize the microbial community structure and functional gene abundance related to nitrogen metabolism, and to reveal the underlying metabolic mechanisms.

## 2. Materials and Methods

### 2.1. Experimental Setup and Water Quality

**Figure 1:**
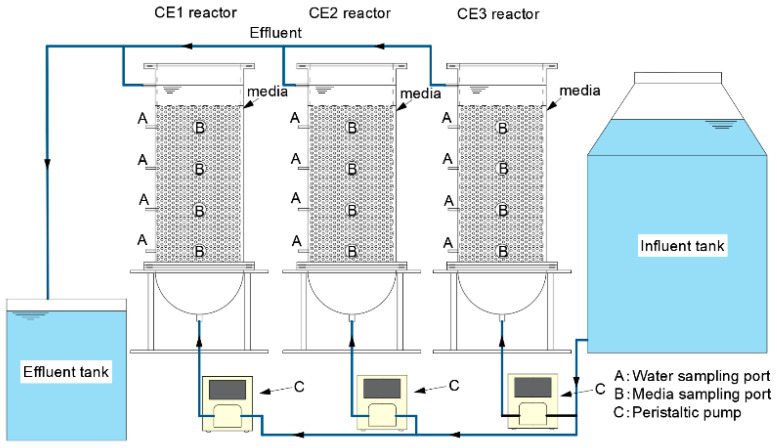
Experimental setup of the DNBF reactors.

The experimental system consisted of three main components: a 500 L influent tank storing synthetic nitrate-contaminated water, three DNBFs for water treatment, and an effluent tank collecting processed water (Figure 1). The influent tank was connected to the DNBFs by a peristaltic pump (Kamoer Fluid Tech, Shanghai, China; model DIP1500), which regulated the influent flow rate. Each DNBF reactor was constructed from 10 mm thick plexiglass and internally divided into three functional zones: influent zone, media zone (filled with ceramsite), and effluent zone. The influent zone, a hemisphere with a 310 mm inner diameter, transitioned into the cylindrical media zone, which was 530 mm high and provided a working volume of 40 L. The media zone was filled with ceramsite media with different pore structures (marked as CE1, CE2, and CE3). The particle size of all three types of ceramsite media was uniformly 4–6 mm. Above the media zone, the effluent zone, measuring 70 mm in height, connected to the effluent tank via an 8 mm inner diameter outlet pipe positioned at its top.

The influent synthetic nitrate-contaminated water was manually prepared by dissolving glucose (181.70 g), sodium nitrate (182.10 g) and potassium dihydrogen phosphate (13.20 g) to serve as carbon, nitrogen and phosphorus sources, respectively, in a 500 L influent tank. Trace elements were derived from the background elements present in on-site well water (Table S1). Throughout the experiment period, the influent concentration of COD_cr_ was approximately 300.00 mg/L, the concentration of NO_3_^−^-N was approximately 60.00 mg/L, and the C/N ratio was approximately 5.

### 2.2. Experimental Methods

#### 2.2.1. Operational Settings

The experiment comprised two phases: the inoculation and cultivation period and the continuous operation period. Three ceramsite media types (CE1, CE2, CE3) were tested in separate DNBF reactors under identical real-environmental conditions. During the operational period the water temperature naturally ranging from 24.5 to 27.9 °C. The influent dissolved oxygen (DO) concentration fluctuated between 2.54 and 3.47 mg/L. Water quality parameters were measured daily throughout 76 days of continuous operation, ensuring statistical validity. The operating parameters for the experiment are detailed in Table S2, while the reagents and experimental instruments required are listed in Table S3.

(1) Inoculation and cultivation period: The denitrifying microbial agent (GanDu, GANDEW-DEN) was used to prepare a 4 g/L seed suspension (free-cell suspension) for inoculation. Peristaltic pumps were utilized to circulate the seed suspension from bottom to top in the DNBFs. Daily water samples were collected at 8 a.m. from three DNBFs, and water indicators NO_3_^−^-N, NO_2_^−^-N, NH_4_^+^-N and COD_cr_ were determined. Carbon and nitrogen sources were supplemented to maintain the COD_cr_ at 300.00 mg/L and the NO_3_^−^-N at 60.00 mg/L. After 3 days, once the seed suspension became viscous and the concentration of NO_3_^−^-N dropped below 5.00 mg/L, the cycle period ended, and the system shifted to continuous operation.

(2) Continuous operation period: This period was divided into the start-up phase (0–17 d) and the loading phase (18–76 d). During the start-up phase, the bacteria on the media surface were predominantly in a reversible adhesion state [26], making bacteria susceptible to being dislodged by water currents. Consequently, a higher hydraulic retention time (HRT) of 4 h was set. According to the Standard for Design of Outdoor Wastewater Engineering (GB 50014-2021, China) [27] and Standard for Design of Upflow Denitrification Filter (T/CUWA 50053-2023, China) [28], the maximum nitrate load of DNBF can reach 1.5–3.0 kg/(m^3^·d), and the HRT should be greater than 45 min. When the HRT = 1 h, the nitrate load of the influent to the DNBF can achieve 1.44 kg/(m^3^·d). Therefore, the HRT was incrementally decreased to 3 h, 2 h, 1.5 h, and finally to 1 h in the loading phase.

#### 2.2.2. Water Sampling and Analytical Methods

Water samples of influent and effluent from the three DNBFs were collected at 8 a.m. The indicators measured included NO_3_^−^-N, NO_2_^−^-N, NH_4_^+^-N, COD_cr_, temperature (T), and dissolved oxygen (DO). Water samples were filtered through a 0.45 μm membrane prior to analysis. All water quality analyses were conducted according to the relevant standards (Table S4).

The nitrate removal efficiency (N_RE_), chemical oxygen demand removal efficiency (COD_RE_), and volumetric denitrification rate (R_vd_) were calculated as follows:

(1) $$\begin{array}{c} N_{RE}=\frac{\left({NO}_{3}^{-}–N_{inf}-NO_{3}^{-}{–N}_{eff}\right)}{NO_{3}^{-}{–N}_{eff}}\times 100\% \end{array}$$

(2) $$TN={NO}_{3}^{-}–N+{NO}_{2}^{-}–N+{NH}_{4}^{+}–N$$

(3) $${COD}_{RE}=\frac{{(COD}_{inf}-{COD}_{eff})}{{COD}_{inf}}\times 100\%$$

(4) $$R_{\nu d}=\frac{0.024\times Q_{d}\times (TN_{inf}-TN_{eff})}{V}$$

where ${NO}_{3}^{-}–N_{inf}$, ${NO}_{2}^{-}–N_{inf}$, $TN_{inf}$, and ${COD}_{inf}$ are the influent ${NO}_{3}^{-}–N$, ${NO}_{2}^{-}–N$, TN, and COD concentrations, respectively (mg/L); $NO_{3}^{-}{–N}_{eff}$, ${NO}_{2}^{-}–N_{eff}$, $TN_{eff}$, and ${COD}_{eff}$ are the effluent ${NO}_{3}^{-}–N$, ${NO}_{2}^{-}–N$, TN, and COD concentrations, respectively (mg/L); $Q_{d}$ is the flow (L/h); V is the practical volume of the filter (L); 0.024 is conversion coefficient calculated as 24 h/d divided by 1000 (conversion mg/(L·d) to kg/(m^3^·d)).

#### 2.2.3. Physicochemical Characteristics of Media

Chemical composition SiO_2_ and Al_2_O_3_ contents were quantified by X-ray fluorescence (XRF; PANalytical Axios MAX; Malvern Panalytical; Almero, The Netherlands) following Methods for Chemical Analysis of Cement (GB/T 176-2017, China) [29]. pH stability was evaluated via 24 h batch leaching (10 g media/100 mL H_2_O, 25 °C) with pH monitored hourly (Mettler Toledo FE28; Zurich, Switzerland). Mechanical strength was tested under ASTM D7012 using an Instron 3369 (1 mm/min loading rate). The above parameters are listed in Table S5. Surface morphology and porosity were examined via SEM (JEOL JSM-7800F; Japan Electron Optics Laboratory; Tokyo, Japan) at 2000× and 600× magnifications. Pore parameters (effective porosity φ, fractal dimension *D*) were quantified from 600× SEM images using Image-Pro Plus (version 6.0) [30,31], with pores classified as optimal (0.5–25 μm), available (>25 μm), and effective [18,19,20,32].Medium porosity was defined by the following equation [33,34]:

(5) $$\varphi =\frac{A_{\mathrm{pore}}}{A_{image}}\times 100\%$$

where $A_{pore}$ is the area of the pores in the SEM, and $A_{image}$ is the area of the SEM.

The fractal dimension (D) of the pores was calculated using the relationship between pore perimeter and area to quantify the roughness of the packing surface using the following equation [30]:

(6) $$\log s(\varepsilon )=(\frac{D}{2})\times \log A(\varepsilon )+C$$

where ε is the measured unit size (μm); s is the perimeter of the pore (μm); A is the area of the pore (μm^2^); and C is a constant.

#### 2.2.4. Biomass Determination

At both the midpoint and the end of each HRT, equal volumes of medium were sampled from the upper, middle, and lower sections of the media zone in each DNBF. These samples were mixed and placed in a beaker, and the biofilm was removed from the media by scraping and magnetic stirring at 1000 r/min. The detached biofilm was then transferred to a centrifuge tube, brought up to a volume of 50 mL, and mixed thoroughly. The 25 mL prepared suspension was used to measure biomass, while the remaining 25 mL was used to determine extracellular polymeric substances (EPS). The biomass was quantified using the gravimetric method [35]. Briefly, the corundum crucible was dried at 105 °C for 8 h and weighed (G_1_, g). Then, the crucible was filled with 25 mL of the suspension, dried at 105 °C for 8 h, and reweighed (G_2_, g). Finally, the media, after biofilm removal, was also dried at 105 °C for 8 h and weighed (G_3_, g). The biomass of media (M, mg/cm^2^) was calculated as follows [36]:

(7) $$M=(G_{2}-G_{1})/(G_{3}\times S\times 10)$$

where S is the specific surface area of the media (m^2^/g), determined by the gas adsorption method; 10 is the unit conversion factor.

#### 2.2.5. Extracellular Enzyme Polymers (EPS)

EPS were extracted using a mild heat method [37]. First, a 25 mL biofilm suspension was transferred to a centrifuge tube and supplemented with physiological saline to the volume of 50 mL. Then, the mixture was heated in a 60 °C water bath for 30 min and subsequently centrifuged at 4000× *g* for 20 min. The supernatant was collected as the EPS. The extracted EPS were filtered through a 0.45 μm filter membrane, and then the protein (PN) was determined using the modified Lowry method [38], with bovine serum proteins as the standard. Polysaccharides (PS) were determined using the Anthrone-sulphate method [39], with glucose as the standard. The total EPS concentration was calculated as the sum of PN and PS concentrations.

#### 2.2.6. High-Throughput Sequencing and Analysis

A total of 12 samples, including three biological replicates of seed sludge and nine spatial biofilm samples (upper, middle, and lower sections from each DNBF reactor), were collected and stored at −80 °C until analysis. All samples were sent to Shanghai Majorbio Bio-pharm Technology Co., Ltd. (Shanghai, China) for 16S rRNA high-throughput sequencing analysis. DNA was extracted from microbial communities using the E.Z.N.A.^®^ Soil DNA Kit (Omega Bio-tek, Norcross, GA, USA), and the concentration and purity were assessed. The V3-V4 region of the 16S rRNA gene was amplified by PCR with universal primers 338F(5′-ACTCCTACGGGAGGCAGCAG-3′)-806R.(5′-GGACTACHVGGGTWTCTAAT-3′). Purified PCR products were used to construct libraries with the NEXTFLEX Rapid DNA-Seq Kit and sequenced on the Illumina MiSeq (PE300) platform. Raw reads were quality-filtered using Fastp software (v0.19.6) to remove low-quality sequences (Q-score < 20, length < 200 bp), and paired-end reads were merged with FLASH (v1.2.11). Sequences with ≥97% similarity were clustered into operational taxonomic units (OTUs) using UPARSE v7.1. Taxonomic annotation was performed with the RDP Classifier (v2.11) against the Silva 138 database, (confidence threshold: 0.7). Alpha diversity indices (Chao1, Shannon, Simpson) were calculated using Mothur (v1.46.1). Beta diversity was analyzed via principal coordinate analysis (PCoA) based on Bray–Curtis distances, and permutational multivariate analysis of variance (PERMANOVA, 9999 permutations) was conducted using the vegan package (v2.6.4) in R (v4.1.2) to assess significant differences between groups. Linear discriminant analysis effect size (LEfSe) was applied to identify differentially abundant taxa (LDA score > 4, *p* < 0.05) from phylum to genus levels.

#### 2.2.7. Statistical Analysis and Visualization

Experimental data were preprocessed, and error checking using Excel 2019 (Microsoft, Redmond, WA, USA) was conducted. Statistical analyses were performed in SPSS (v26.0, IBM, Armonk, NY, USA), with one-way ANOVA and Tukey’s post-hoc test for parametric data (normality confirmed by Shapiro–Wilk test, α = 0.05) and Kruskal–Wallis test with Dunn–Bonferroni correction for non-parametric data. Microbial community composition at the phylum and genus levels was visualized as stacked bar charts in OriginPro (v2021, OriginLab, Northampton, MA, USA). Functional predictions for nitrogen metabolism were conducted using PICRUSt2 (v2.4.1, Harvard University, Cambridge, MA, USA) with default settings, referencing the Kyoto Encyclopedia of Genes and Genomes (KEGG) database (Release 2021.10). KEGG Orthology (KO) annotations were mapped to nitrogen metabolism pathways (map00910), and functional gene abundance plots were generated using OriginPro 2021 (v2021, OriginLab, Northampton, MA, USA). Metabolic pathway schematics were reconstructed in Microsoft Visio (v2019, Microsoft, Redmond, WA, USA) based on KEGG annotations. Relationships between ceramsite pore structure, biofilm characteristics (biomass, EPS), microbial communities, and denitrification performance were investigated via partial least squares path modeling (PLS-PM) using the plspm package in R (v4.4.2, R Foundation for Statistical Computing, Vienna, Austria), with path coefficients, explained variance (*R*^2^), and goodness-of-fit indices calculated to validate the model.

## 3. Results and Discussion

### 3.1. Pore Structure Characteristics of Media

#### 3.1.1. Media Surface Morphology

**Figure 2:**
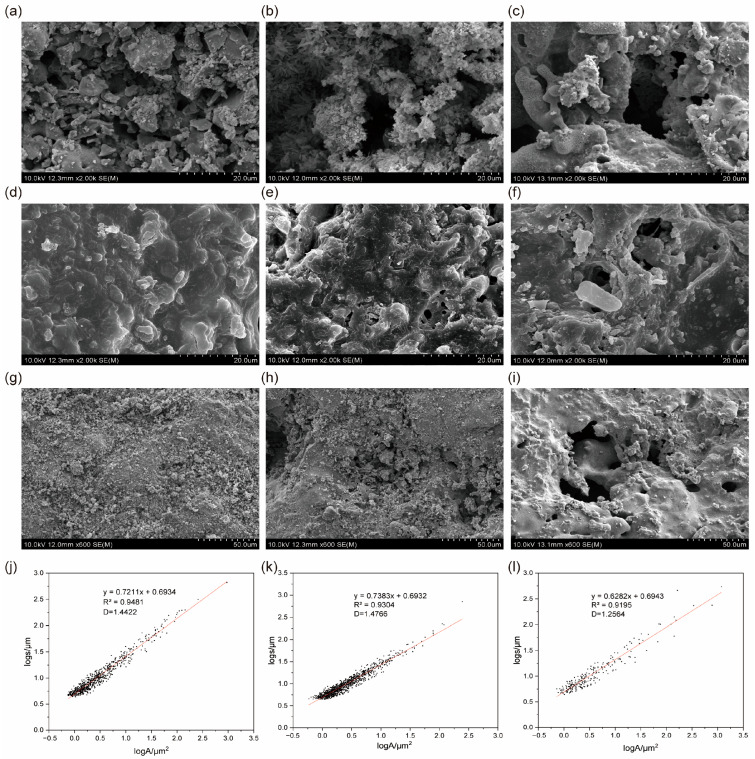
SEM characterization of ceramsite surface morphology evolution and pore structure dynamics. (**a**–**c**) Pre-biofilm surface morphology (2000×): CE1 (**a**), CE2 (**b**), and CE3 (**c**); (**d**–**f**) Post-biofilm surface morphology (2000×): CE1 (**d**), CE2 (**e**), and CE3 (**f**); (**g**–**i**) Pore structure analysis raw SEM images (600×): CE1, CE2, and CE3; (**j**–**l**) Fractal dimension correlations between pore perimeter and area based on SEM images (600×): CE1 (**j**), CE2 (**k**), and CE3 (**l**).

Before biofilm formation, the surface of CE1 was characterized by uniform, shallow, small pores and a relatively rough morphology (Figure 2a). The surface of CE2 featured both uniform shallow pores and scattered deeper, large pores, with a rougher substrate surface (Figure 2b), indicating a complex and functional pore structure. In contrast, the surface CE3 was dominated by deeper, larger pores, and had a smoother substrate (Figure 2c). In summary, CE1 and CE2 had more densely packed small pores than CE3. These differences in pore characteristics may impact biofilm growth [40].

After biofilm formation, the surfaces of CE1 and CE2 were densely covered with biofilms, obscuring the underlying substrate (Figure 2d,e). Notably, the biofilm of CE2 included fine pores, possibly a result of the larger pores presented on its substrate. In contrast, CE3 exhibited a markedly lower biofilm attachment density, with biofilms mainly confined within the pores, resulting in some surface areas remaining biofilm-free (Figure 2f). This is consistent with studies indicating that a dense, uniform small pore size fosters even biofilm distribution on media surfaces [41,42].

#### 3.1.2. Media Porosity and Fractal Characteristics

The pore characteristics of three ceramsite media were quantified through SEM image analysis (Figure 2g–i). The surface pore characteristics of the three media are summarized in Table 1. CE2 exhibited the highest effective porosity (>0.5 μm) at 55.8%, surpassing both CE1 with 47.8% and CE3 with 46.7%. The surface optimal porosity (0.5–25 μm) for CE1 and CE2 were 47.9% and 47.8%, respectively, which was much higher than CE3 (23.5%). However, CE3 had the highest available porosity (>25 μm) at 23.2%, significantly greater than CE1 and CE2. The presence of micropores accessible to microbes is crucial for biofilm formation [43,44]. Enhancing the effective porosity, particularly the optimal pore size range that maximizes microbial accumulation, promotes biomass accumulation on the media surface. In conclusion, the pore characteristics of CE2 were most conducive to microbial attachment, followed by CE1.

Analysis of 600× SEM images (Figure 2a–c) revealed the pore fractal dimensions of the three ceramsite types, showing a strong linear correlation between the microscopic pore perimeter and area (R^2^ > 0.91; Figure 2d–f), indicative of the fractal nature of the pore structures on the medium surfaces. The fractal dimensions of the three ceramsites are shown in Table 1. As a measure of surface roughness [45], the fractal dimension of CE2 is slightly higher than that of CE1, while CE3 has the lowest value. As fractal dimension typically increases with porosity and decreases with pore size [46,47], this is consistent with our findings that CE1 and CE2, having higher optimal porosity, also exhibited higher fractal dimensions compared to CE3. CE2, with higher effective porosity, displayed a higher fractal dimension and greater surface roughness. Surface roughness is known to enhance microbial growth conditions [33]. In conclusion, the higher pore fractal dimension of CE2 suggests a rougher surface, potentially more conducive to biofilm attachment.

### 3.2. The Performance of DNBFs with Different Pore Structure Media

**Figure 3:**
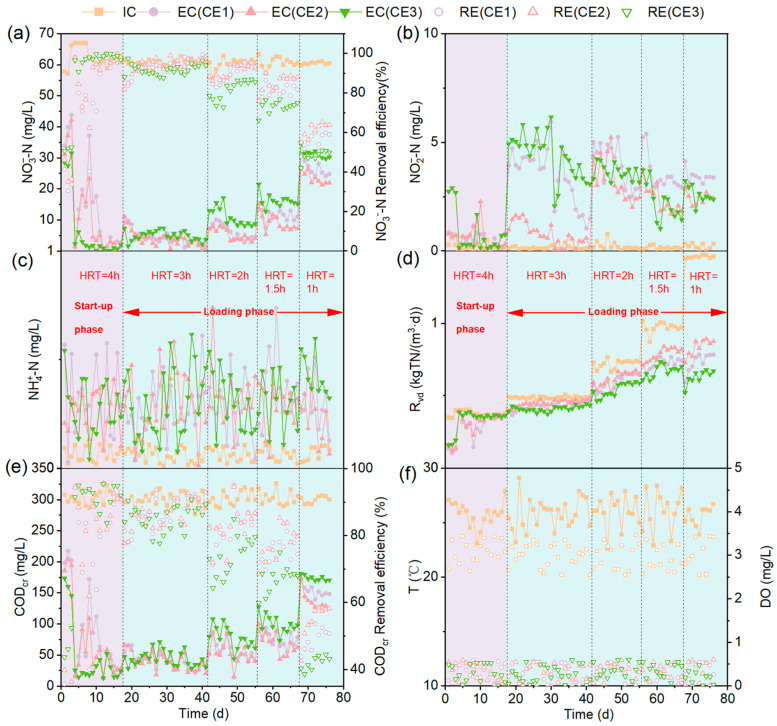
Effect of packing pore structure and HRT on pollutant removal effect. (**a**) NO_3_^−^-N, (**b**) NO_2_^−^-N, (**c**) NH_4_^+^-N, (**d**) R_vd_ (TN), (**e**) CODcr, and (**f**) Temperature T (°C) versus change in dissolved oxygen (DO). IC: influent concentration; EC: effluent concentration; RE removal rate.

The dynamic performance of the DNBFs under varying HRTs and ceramsite pore structures is comprehensively illustrated in Figure 3, which compares six key parameters: NO_3_^−^-N, NO_2_^−^-N, NH_4_^+^-N, Rvd, COD_cr_, and temperature-DO. Table S7 provides a detailed comparison of DNBFs’ denitrification capabilities across different HRTs. During the start-up phase, the NO_3_^−^-N removal efficiency (N_RE_) across the three DNBFs was relatively low during 0–3 days, varying from 35.15% to 51.72% (Figure 3a). The N_RE_ gradually improved over time, with CE3 reaching 90% by day 5, CE2 and CE1 by days 9 and 11, respectively. The NO_2_^−^-N concentrations in the effluents remained generally low, with CE3 showing slightly higher levels (2.78 ± 0.07 mg/L) compared to CE1 (0.80 ± 0.07 mg/L) and CE2 (0.71 ± 0.12 mg/L) from 0 to 3 days. Minor fluctuations in NO_2_^−^-N were observed in all DNBFs between days 8 and 10, followed by stable concentrations below 1.00 mg/L from days 11 to 17 (Figure 3b), indicating a progressive stabilization of the systems. Similarly, NH_4_^+^-N concentrations were maintained at low levels (<0.4 mg/L; Figure 3c), demonstrating the effectiveness of DNBFs in controlling NH_4_^+^-N accumulation. The volumetric denitrification rate (R_vd_) increased and stabilized at 0.35 kg TN/(m^3^·d) for CE1, CE2, and CE3 by days 11, 9, and 5, respectively (Figure 3d), suggesting the biofilm colonization on media [48]. The COD_cr_ concentrations ranged from 18.48 to 26.47 mg/L. CE3 achieved the highest COD_cr_ removal rate (COD_RE_; 93.84%), followed by CE2 (91.43%) and CE1 (91.17%) (Figure 3e; Table S3). These results indicate that the larger available porosity of CE3 facilitated earlier biofilm formation, thus accelerating the activation of DNBFs.

During the loading phase, the N_RE_ of three DNBFs gradually decreased as the HRT reduced from 3 h to 1 h (Figure 3a). This is attributed to the escalating influent NO_3_^−^-N loading, suggesting an upper limit to the removal capacity of DNBFs [49]. CE1 and CE2 initially showed higher N_RE_ than CE3, especially as the HRT decreased to 1.5 h and 1 h, with CE2 demonstrating superior performance likely due to its rougher surface facilitating greater biofilm accumulation [50]. Notably, only the effluent NO_3_^−^-N of CE2 (7.28 mg/L) complied with the Standards for Drinking Water Quality (GB 5749-2022, China; NO_3_^−^-N ≤ 10 mg/L, NO_2_^−^-N ≤ 1 mg/L, NH_4_^+^-N ≤ 0.5 mg/L) [51] when HRT = 1.5 h. NO_2_^−^-N and NH_4_^+^-N were detected in the effluents of all DNBFs (Figure 3b,c). The accumulation of NO_2_^−^-N may be due to a higher efficiency in electron acquisition of NO_3_^−^-N during the reduction process, coupled with a lower synthesis capacity of nitrite reductase compared to nitrate reductase [52,53]. NH_4_^+^-N may be generated from the dissimilatory nitrate reduction to ammonium (DNRA) process [54]. R_vd_, a direct indicator of denitrification performance, increased as HRT decreased (Figure 3d). Below an HRT of 2 h, the R_vd_ trend was CE2 > CE1 > CE3, with more significant differences as HRT decreased to 1.5 h (*p* < 0.01, η^2^ = 0.925). Under the condition of ensuring the effluent NO_3_^−^-N meets the GB 5749-2022 [51], the maximum R_vd_ of CE2 is 0.82 kg/(m^3^·d). Liu et al. fabricated ceramsite fillers with large porosity through material composition adjustments and examined HRT effects on denitrification. A nitrate removal efficiency of 79.9% was achieved at 20.0 mg/L influent nitrate and 0.5 h HRT, and the R_vd_ was 0.77 kg/(m^3^·d), which was lower than the maximum R_vd_ for the CE2 ceramsite filler in this study [25]. This suggests that R_vd_ is influenced by the pore structure of the medium, with the highest effective porosity and the higher ratio optimal porosity of CE2 leading to the best denitrification performance. Although CE1 and CE3 had similar effective porosity, the higher optimal porosity ratio in CE1 may explain why its R_vd_ was higher than CE3. The COD_RE_ of the DNBFs decreases with decreasing HRT, especially at HRT = 1 h (Figure 3e). CE2 had the highest COD_RE_, followed by CE1, with CE3 being the lowest. This aligns with the R_vd_, indicating that the superior micro-pore structure of CE2 is effective for NO_3_^−^-N and COD_cr_ removal. However, the effluent COD_cr_ concentration did not meet the GB 5749-2022 [51] during continuous operation. In practical applications, adjusting the C/N ratio [55] and incorporating aeration post-DNBFs [56] could improve the performance. Three DNBFs effectively removed DO, with the effluent DO concentrations below 0.5 mg/L. The water temperature, ranging from 24.5 to 27.9 °C, was optimal for the denitrification bacteria activity [57]. Overall, CE2 with high effective porosity (55.8%) and optimal porosity ration (83.1%) exhibited the best performance at an HRT of 1.5 h by balancing denitrification performance with economic feasibility.

The superior denitrification performance of CE2 not only enhanced treatment efficiency but also presented promising economic advantages for large-scale applications. Compared with CE1 and CE3 ceramsite media, CE2 enabled 9.8% and 17.1% reduction in reactor volume under the same treatment load when HRT = 1.5 h, significantly lowering capital costs for infrastructure construction. The high R_vd_ (0.82 kg TN/(m^3^·d) at HRT = 1.5 h) reduced energy consumption by minimizing HRT and pumping requirements, translating to 15–20% savings in operational costs compared to media with lower efficiency. From a manufacturing perspective, ceramsite is a mature industrial product with established production chains, and the adjustment of pore structure (e.g., controlling the optimal porosity ratio) can be achieved through standardized firing processes, avoiding excessive costs for specialized materials. The rough surface and high fractal dimension of CE2 promote robust biofilm adhesion, reducing the frequency of media replacement and backwashing—key maintenance expenses in biological filters. Life cycle analysis suggests that CE2’s service life (5–8 years) is 1.5 times longer than conventional media due to its resistance to biofilm detachment under hydraulic stress, further decreasing long-term maintenance costs. Moreover, CE2 aligns with stringent nitrate discharge regulations (e.g., GB 5749-2022) [51] by achieving effluent NO_3_^−^-N concentrations of 7.28 mg/L, potentially avoiding penalties for non-compliance and reducing the need for post-treatment steps. While the initial material cost of CE2 is slightly higher than quartz sand (≈10–15% increase), the overall cost-benefit ratio is favorable due to its high efficiency and low operational demands. This makes CE2 a cost-effective choice for municipal and industrial wastewater treatment systems, particularly in regions facing strict nitrogen emission targets.

It should be noted that this study utilized synthetic wastewater to systematically isolate the effects of HRT and media pore characteristics on denitrification performance. While synthetic systems simplify influent composition to decouple target parameters from real-world confounding factors (e.g., fluctuating nutrient loads, inhibitory substances), translating these findings to practical applications requires addressing three critical challenges: (1) microbial community dynamics, where indigenous populations in real wastewater—such as competitive nitrifiers or synergistic fermentative bacteria—may alter biofilm structure and denitrification efficiency; (2) inhibitory contaminants (e.g., heavy metals, antibiotics, high ammonia), which may necessitate pre-treatment strategies (e.g., adsorption, chemical oxidation) to mitigate toxicity and enhance biofilm resilience; and (3) nutrient variability, requiring adaptive control strategies (e.g., real-time C/N ratio adjustment) to maintain efficiency under fluctuating carbon/nitrogen loads. Despite these complexities, the controlled conditions here provide a robust theoretical framework for optimizing medium design, with future studies recommended to validate performance in real wastewater systems. Despite the rigorous quality control throughout the experimental design and operation, potential sources of error and outliers still warrant consideration. For example, minor inaccuracies in manual wastewater preparation—specifically in solute weighing and volumetric dilution—led to slight deviations from the target influent NO_3_^−^-N concentrations during the first 6 days of the start-up phase, and some fluctuations observed in the loading phase. Environmentally, subtle temperature and DO fluctuations might have influenced microbial activity, particularly during HRT transitions, as microorganisms required time to acclimate, potentially causing data variability. In future studies, increasing the sample size and using more precise equipment will help mitigate the impact of errors and outliers on the results. Additionally, validating the proposed HRT and media design criteria under real wastewater conditions is critical, with a focus on long-term biofilm stability monitoring and carrier fouling mitigation strategies tailored to dynamic influent compositions. While this study compared ceramsite media with distinct pore structures, incorporating traditional substrates (e.g., quartz sand) as controls in future work will further validate the efficacy of pore optimization for denitrification efficiency.

### 3.3. Biomass and EPS Analysis

**Figure 4:**
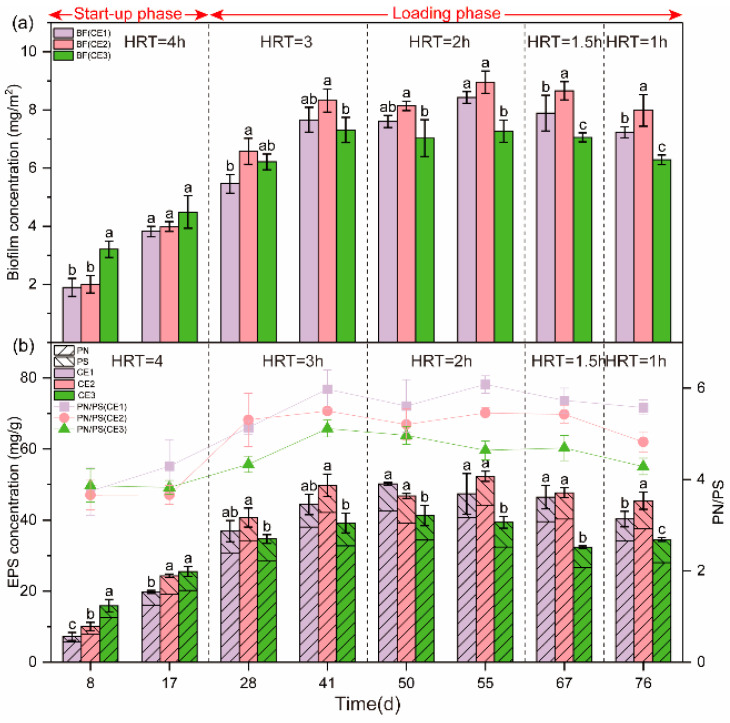
Variation of biofilm biomass and EPS content of different media over time under various HRT. (**a**) Biomass concentration of three media. (**b**) EPS concentration and the PN/PS ratio. Mean ± standard deviations (*n* = 3) are shown. Different lowercase letters show statistically significant differences between media treatments within the same time (*p* < 0.05).

The biofilm biomass development can be categorized into three distinct phases: a rapid growth period (0–41 days), a sloughing and updating period (41–55 days), and a maturation phase (55–76 days; Figure 4), aligning with prior research [58]. In the rapid growth period, coinciding with the start-up phase and the pre-loading phase (HRT = 3 h), microorganisms gradually adapted to the environment, and biomass accumulated rapidly under nutrient-sufficient conditions. During the sloughing and updating period (HRT = 2 h), the biomass of the media approached the system’s carrying capacity, its growth rate slowed significantly due to limited substrate and space for proliferation. During the maturation period (HRT = 1.5 h, 1 h), biomass stabilized near the medium’s carrying capacity limit, with a minor decrease likely due to increased water flow shear forces [59].

CE3 exhibited the highest biomass during the early rapid growth period (0–17 days), being 18.4% and 12.5% higher than CE1 and CE2, respectively (Figure 4). The biofilm initially accumulates in larger pores (59–69 μm) [60], and the larger pore sizes in ceramsite ring media (50 μm–1.3 mm and 5–25 μm) led to shorter start-up times due to lower flow velocities, which are conducive to microbial deposition during early biofilm formation [61]. This explains why CE3 reached a steady state in NO_3_^−^-N effluent earlier in the start-up phase (Figure 3a). In contrast, during the later rapid growth period (17–41 days), microorganisms transitioned from reversible adsorption to irreversible adhesion on the media, driven by the cohesive force of EPS. Biomass of CE2 and CE1 surpassed CE3, exhibiting as CE2 (8.3 mg/m^2^) > CE1 (7.7 mg/m^2^) > CE3 (7.3 mg/m^2^). This was attributed to the higher optimal porosity of CE2 and CE1, and the larger effective porosity of CE2, which provides more sites for microbial attachment.

**Figure 5:**
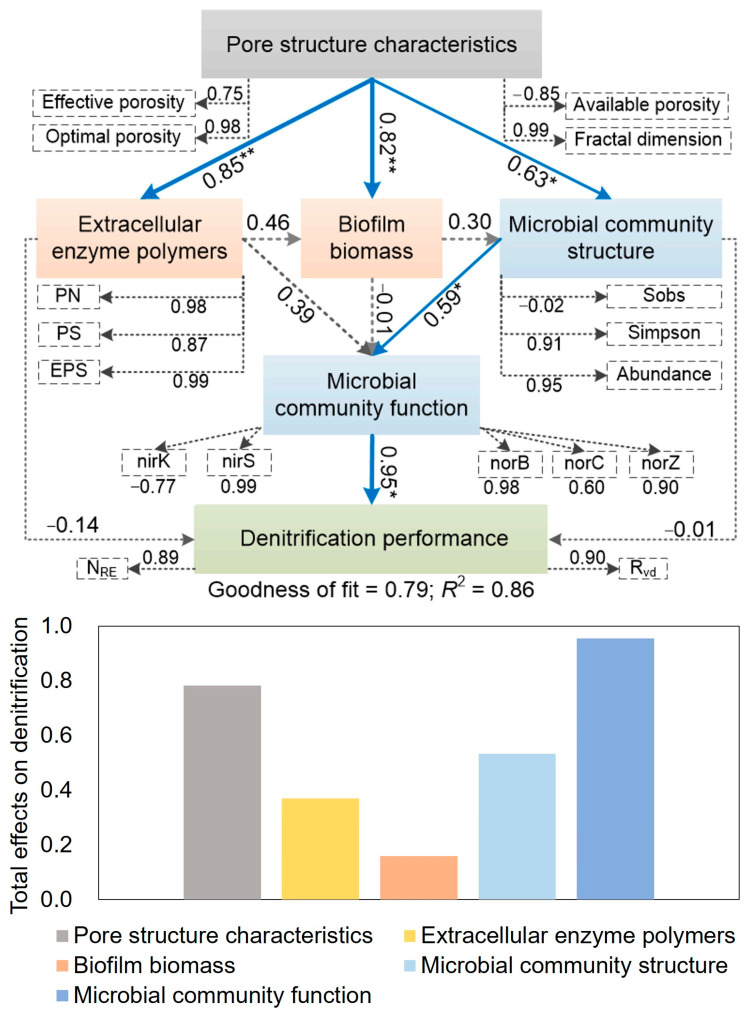
Partial least squares path model (PLS-PM) estimates of the impact of pore structure of ceramsite on biofilm biomass, extracellular enzyme polymers, and microbial community characteristics, and the denitrification performance in denitrification biological filters. Each box denotes a latent or measured variable. The dashed rectangles represent the loading for the latent variables. The widths and numeric data of the arrows represent the standard path coefficients (* *p* < 0.05, ** *p* < 0.01). The blue and red indicate statistically significant positive and negative correlations (*p* < 0.05), respectively, whereas grey lines illustrate the insignificant correlation. The *R*^2^ value represents the explained variance. PN: protein; PS: polysaccharides; EPS: PN + PS; Abundance: the relative abundance of denitrifying bacteria; N_RE_: nitrate removal efficiency; R_vd_: volumetric denitrification rate.

During the sloughing and updating period (HRT = 2 h), the biomass exhibited CE2 (9.0 mg/m^2^) > CE1 (8.4 mg/m^2^) > CE3 (7.3 mg/m^2^). In the maturity period (HRT = 1.5 h, 1 h), the order remained the same, with CE2 (8.5 mg/m^2^) leading, CE1 (7.8 mg/m^2^) next, and CE3 (6.9 mg/m^2^) last. The biomass accumulation correlated with the effective and optimal porosity of the media. The effective porosity of CE1 and CE3 is similar, but the percentage of optimal porosity in CE1 is higher than that of CE3. The optimal porosity of CE1 and CE2 is comparable, yet the effective porosity of CE2 is higher than that of CE1. The biomass ranking correlates with the trends in effective and optimal porosity. Specifically, higher effective porosity—particularly optimal porosity—promotes biomass accumulation. Therefore, enhanced effective porosity, especially optimal porosity, promotes biomass accumulation. These results indicate that the pore structure significantly influences biomass growth and distribution, which was supported by PLS-PM (standard path coefficient = 0.82, *p* < 0.01; Figure 5). The increased biofilm biomass improved denitrification performance (total effects = 0.16; Figure 5).

**Figure 6:**
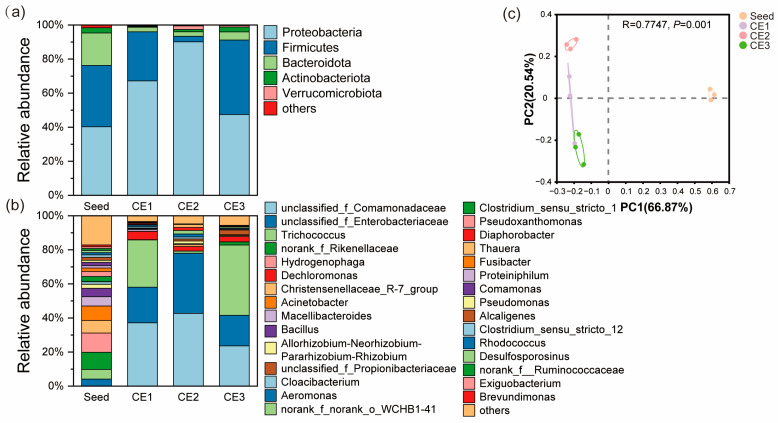
Relative abundance and principal coordinate analysis (PCoA) of seed suspension and media biofilm communities. (**a**) phylum-level communities; (**b**) genus-level communities; (**c**) PCoA analyses.

The EPS within the biofilm exhibited analogous patterns of variation, with rapid increases during the initial start-up phase and the early loading phase (HRT = 3 h), followed by a deceleration in growth rate (HRT = 2 h), culminating in stabilization (HRT = 1.5 h, 1 h; Figure 6). This is attributed to the positive correlation between EPS and biomass [59]. Initially, EPS content ranked as CE3 > CE2 > CE1, possibly due to high biomass promoting EPS secretion [62]. During the loading phase, the ranking shifted to CE2 > CE1 > CE3. When EPS levels stabilized (55–76 days), significant differences among the DNBFs were noted (*p* = 0.00, η^2^ = 0.63), with CE2 (48.5 mg/g) exceeding CE1 (44.7 mg/g) and CE3 (35.4 mg/g). PLS-PM also indicated that pore structure characteristics positively affected the EPS content (standard path coefficient = 0.85, *p* < 0.01; Figure 5). EPS is known to impact biofilm formation and nitrogen removal [8,14,62], which likely explains the variations in denitrification efficiency observed among the DNBFs (total effects = 0.37; Figure 5).

PN/PS is an important indicator of biofilm activity and aggregation properties [63]. In this study, the three types of media exhibited PN/PS exceeded 1, suggesting that the biofilms possessed good aggregability and high activity [64]. During the start-up phase and early loading phase (HRT = 3 h), the PN/PS of all DNBFs increased (Figure 6), which likely facilitated biomass stabilization and enhanced microbial interactions, thereby enhancing denitrification efficiency [9]. The PN/PS of three DNBFs slightly declined with the decrease in HRT (2 h to 1 h), possibly due to increased water shear stimulating microorganisms to produce more PS, which enhanced the adhesion of the biofilm to media [65]. During the start-up phase, the PN/PS of the three media showed no significant differences, likely due to microorganisms focusing on survival and reproduction while acclimating to the new environment. The uniform nutrient supply across the three DNBFs further contributed to similar microbial growth and metabolic activity.

However, PN/PS ranked as CE1 > CE2 > CE3 during the loading phase. The pore structure of the media played a role in the secretion of PN and PS [15,66]. CE3, with the lower fractal dimension and surface roughness, required more PS to enhance biofilm adhesion. On the contrary, CE1 and CE2 showed better biofilm aggregation and activity, making them more favorable for denitrification in the filters.

### 3.4. Microbial Community Analysis

#### 3.4.1. Alpha Diversity Analysis

Sobs, Chao, ace, Shannon, Simpson, and Coverage indices were selected to characterize the microorganisms in the seed suspension and the biofilm on media. Specifically, Sobs, Chao 1, and Ace indices reflect the richness of the microbial community, while the Shannon and Simpson indices evaluate its diversity. The Coverage index was used to assess the completeness of the sample library.

The results showed that the microbial richness and diversity in the seed suspension were significantly higher than in the media biofilms (Table S8). This disparity is attributed to natural selection and competition within specific culture environments, which favor bacteria adapted to those conditions, leading to reduced diversity. A similar phenomenon was reported in [67], which examined sequencing batch biofilm reactors for the treatment of wastewater with low C/N. In addition, there were no significant differences in bacterial richness and Shannon’s index among the three media biofilms (*p* > 0.05). CE2 had a significantly higher Simpson’s index compared to CE3 and the seed suspension, indicating a more dominant bacterial community in CE2. Finally, the Coverage index for all samples exceeded 99.80%, demonstrating high library coverage and reliable results.

#### 3.4.2. Bacterial Community Composition

The dominant phyla (relative abundance > 1%) across all samples included *Proteobacteria*, *Firmicutes*, *Bacteroidota*, and *Actinobacteriota*, which collectively accounted for 97.3–99.1% of the total sequenced reads (Figure 6a). These dominant phyla were also identified in denitrification systems in previous studies by Yi et al. [68] and Liao et al. [69]. Among these, the phylum with the highest relative abundance in all samples was *Proteobacteria* (40.3–90.1%). Notably, *Proteobacteria*, a key denitrifying bacterial group [8,53], was enriched in the biofilms of three media, with the highest abundance observed in CE2 (90.1%), followed by CE1 (67.2%) and CE3 (47.4%). This enrichment of *Proteobacteria* suggests a strong denitrification capacity of DNBFs. *Firmicutes*, the second most abundant phylum capable of degrading macromolecular organic matter, was less consistent, with 36.0% in the seed suspension, peaking at 43.7% in CE3, and lower in CE2 (3.2%) and CE1 (28.7%). This variation may be attributed to the higher residual COD_cr_ in CE3, requiring more *Firmicutes* for degradation [54]. Additionally, *Bacteroidota* and *Actinobacteriota*, known for degrading macromolecular organic matter, provide essential support for bacterial survival in environments with excessive NO_3_^−^-N [54]. In this study, the relative abundance of *Bacteroidota* and *Actinobacteriota* in the seed suspension was higher than in the three media biofilms, with *Bacteroidota* ranging from 2.7% to 4.8% and *Actinobacteriota* from 0.4% to 2.9%. The differences in dominant phyla abundance likely contribute to the varying denitrification efficiency among the DNBFs.

At the taxonomic level (with a focus on genera), the differences in microbial communities among samples were more pronounced (Figure 6b). The seed suspension had 20 dominant genera (relative abundance > 1%), while CE1, CE2, and CE3 biofilms had 3, 8, and 2, respectively. The reduced dominance in media biofilms compared to seed suspension likely resulted from environmental selection under experimental conditions. CE2 exhibited greater diversity in both genera and unclassified families than CE1 and CE3, possibly due to its higher effective porosity and complex pore structure, which offer varied bacterial habitats. Several taxa identified in this study are reported denitrifying bacteria, including unclassified_f_Comamonadaceae, unclassified_f_Enterobacteriaceae, and *Dechloromonas* [48,70,71]. In CE2, unclassified_f_Comamonadaceae (42.7%) and unclassified_f_Enterobacteriaceae (35.3%) were significantly more abundant than in CE1 and CE3. The unclassified_f_Comamonadaceae contains genes for complete denitrification, reducing NO_3_^−^-N to N_2_ [72]. The peritrichous flagella of unclassified_f_Enterobacteriaceae enhance surface attachment, promoting high biomass on CE2 and the reduction of NO_3_^−^-N to NO_2_^−^-N [70]. Conversely, *Trichococcus* was more abundant in CE3 (41.3%) and CE1 (27.8%) than in CE2 (1.3%), and is known to decompose glucose into acetic acid and ethanol, which support denitrification [65]. Other heterotrophic denitrifying bacteria identified included *Aeromonas* [73], *Thauera* [74], and *Diaphorobacter* [75]. *Aeromonas* reduces NO_3_^−^-N to NO_2_^−^-N [73], while *Propionibacteriaceae* [76] and *WCHB1-41* [77] contribute to organic matter degradation. Overall, CE2 had the highest relative abundance of dominant denitrifying bacteria (85.7%), followed by CE1 (62.9%) and CE3 (44.6%), likely due to thicker biofilms of CE2 forming anoxic zones and adsorbing organic matter, thus enhancing denitrification.

Principal coordinate analysis (PCoA) of Bray–Curtis distances revealed distinct clusters of bacterial genera between the seed suspension and the three media, with clear separation between groups (Figure 6c). The seed suspension and media samples were markedly distant along the first principal component, and the media samples maintained significant differences along the second principal component (R = 0.78, *p* = 0.001), indicating significant differences in microbial communities across biofilms with distinct pore structures.

**Figure 7:**
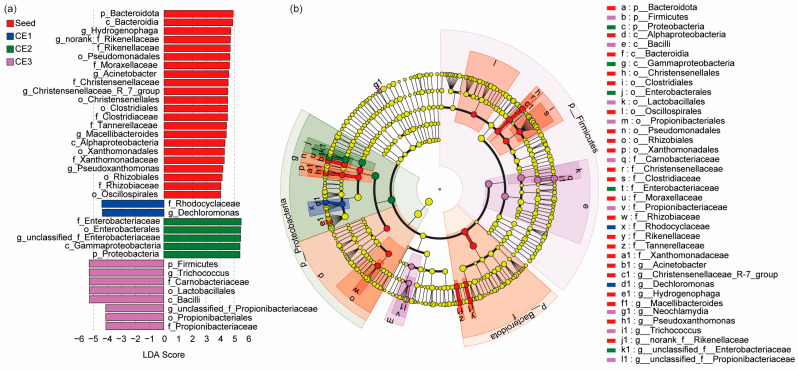
Lefse analysis. (**a**) Linear discriminant analysis (LDA) histograms; (**b**) Lefse evolutionary branching plots of the distribution of biomarkers with significant differences from phylum to genus level in seed suspension and three media communities.

To further identify core differential organisms, Lefse analysis was performed from the phylum to genus level (Figure 7). Biomarker species with LDA scores above 4 were identified in the seed suspension and media samples (Figure 7a). The seed suspension featured biomarkers like *Clostridiaceae*, *Rikenellaceae*, and *Christensenellaceae_R_7_group*, which are linked to anaerobic fermentation, converting carbohydrates and proteins into organic acids [78,79]. *Hydrogenophaga*, a primary participant in hydrogen autotrophic denitrification, was also identified [80]. Other biomarkers included *Acinetobacter*, which performs heterotrophic denitrification [8], and *Pseudoxanthomonas*, which combines heterotrophic denitrification with phosphorus removal [81]. In the media, CE1 harbored *Dechloromonas*, a heterotrophic denitrifier, while CE2 was characterized by unclassified_f_Enterobacteriaceae, a key denitrifier. CE3 contained *Trichococcus*, related to fermentative decomposition of organic matter, and *Propionibacteriaceae*, contributing to denitrification. Overall, the seed suspension exhibited the highest biomarker species and functional diversity, whereas the media biofilms exhibited reduced diversity. CE1 and CE2 were dominated by heterotrophic denitrifying bacteria, while CE3 hosted a mix of denitrifying and fermentative bacteria. This combination may have diluted the denitrification function in CE3, resulting in the highest denitrification efficiency in CE2 and the lowest in CE3. PLS-PM also supported this point, that higher effective and optimal porosity as well as the fractal dimension enhanced the microbial diversity and the relative abundance of denitrifying bacteria (standard path coefficient = 0.63, *p* < 0.05; Figure 5).

The study employed a stepwise HRT reduction from 3 h to 1 h, with the initial 4-h HRT critical for biofilm establishment. Although microbial dynamics across HRTs were unmonitored, biomass and EPS trends offer insights. During start-up (HRT = 4 h), CE3 showed higher initial biomass (Figure 4a), likely due to its larger pores facilitating low-flow microbial deposition [82]. As HRT decreased to 2 h, CE2 and CE1 surpassed CE3, attributed to their optimal porosity (0.5–25 mm) supporting stable biofilm growth under hydraulic shear [83].

CE2’s stable EPS (48.5 mg/g at HRT = 1 h; Figure 4b) and PN/PS > 1 (Figure 4b) suggest the biofilm’s resilience to flow, possibly via EPS-mediated adhesion [84]. Final sequencing (HRT = 1 h) showed that CE2 harbored a more diverse denitrifying community (e.g., unclassified_f_Comamonadaceae) than CE3 (Figure 6b), likely reflecting initial long-HRT niche formation followed by hydraulic selection. However, direct HRT-microbial succession evidence requires time-series profiling, a limitation to address in future studies.

#### 3.4.3. Functional Prediction of Microbial Communities Based on the KEGG

**Figure 8:**
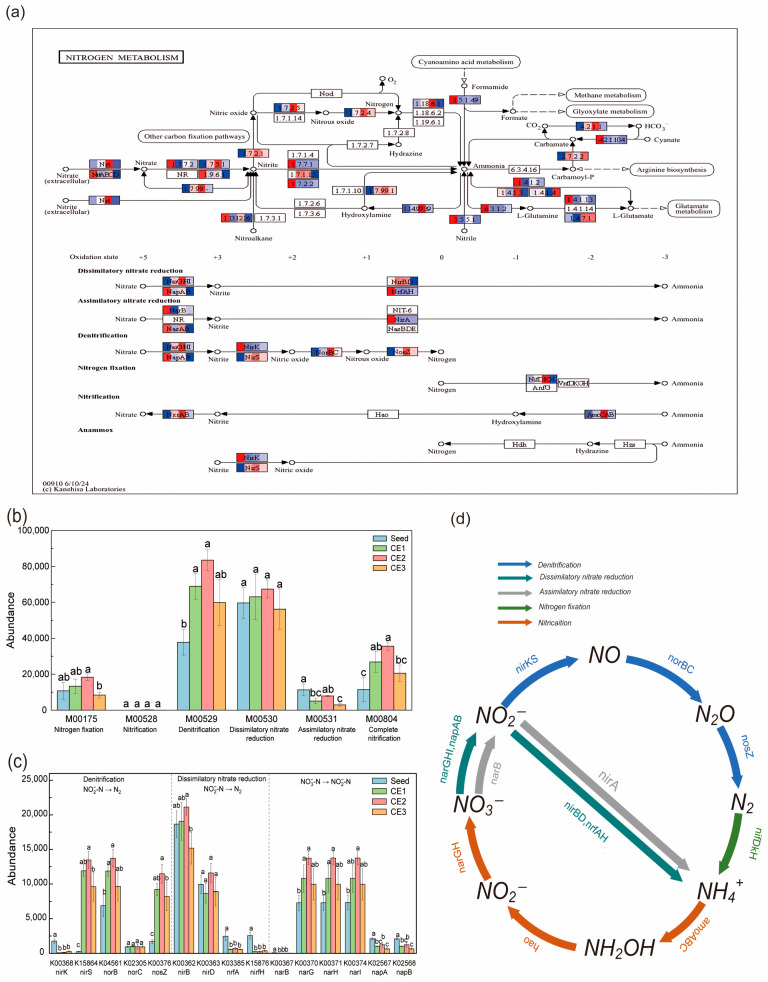
Nitrogen metabolism: function abundance and pathway of seed suspension and medium biofilm. (**a**) Nitrogen-related gene mapping to KEGG; (Note: four squares from left to right represent Seed, CE1, CE2 and CE3); (**b**) Nitrogen metabolism related module abundance; (**c**) Nitrogen metabolism related KO abundance; (**d**) Nitrogen conversion pathway map; Mean ± standard deviations (*n* = 3) are shown. Different lowercase letters show statistically significant differences between media treatments within the same time (*p* < 0.05).

A total of 45 KOs related to nitrogen metabolism were identified in the seed suspension and three types of media biofilms, with 26 KOs associated with six complete nitrogen metabolism modules (Figure 8a), including nitrogen fixation, nitrification, denitrification, dissimilatory nitrate reduction, assimilatory nitrate reduction, and complete nitrification. The denitrification and dissimilatory nitrate reduction modules, involving nitrate reduction, were the most abundant. In the seed suspension, the denitrification module was less abundant than the dissimilatory nitrate reduction module. However, the abundance of the denitrification module in the three media biofilms increased by 82.4% to 121.0% compared to the seed suspension, becoming the most abundant nitrogen metabolism module (Figure 8b). In contrast, the dissimilatory nitrate reduction module showed a smaller increase in abundance (−5.9% to 29.6%) across the media biofilms, making it the second most abundant among the nitrogen converting modules.

A detailed analysis of functional genes related to NO_3_^−^-N reduction (Figure 8c,d) revealed that both denitrification and dissimilatory nitrate reduction pathways can reduce NO_3_^−^-N to NO_2_^−^-N. This process involves genes such as *narGHI* and *napAB*. Subsequently, NO_2_^−^-N can either be reduced to N_2_ by denitrification genes such as *nirKS*, *norBC*, and *nosZ*, or converted to NH_4_^+^-N by dissimilatory nitrate reduction genes such as *nirBD* and *nrfAH*. Notably, the abundance of *nirKS*, *norBC*, and *nosZ* genes significantly increased in the media biofilms (148.3–194.7%), while the abundance of *nirBD* and *nrfAH* genes showed minimal changes (−25.6% to 1.1%). This indicates an enhancement of denitrification and a suppression of dissimilatory nitrate reduction functions in the biofilms, leading to lower NH_4_^+^-N concentrations in the DNBFs effluent (Figure 2c) This aligns with other studies on denitrification with glucose as a carbon source [54], due to the low C/N ratio of the influent [85]. Additionally, the media biofilms showed a higher abundance of *nirKS*, *norBC*, and *nosZ* genes than *nirBD* and *nrfAH*, confirming that denitrification was the primary pathway for NO_3_^−^-N reduction. The *nirKS* genes encode nitrite reductases that catalyze the reduction of NO_2_^−^-N to NO. In the subsequent step, the *norBC* genes encode subunits of nitric oxide reductase, which mediates the conversion of NO to N_2_O. Finally, the *nosZ* gene encodes nitrous oxide reductase, driving the terminal reaction of denitrification by reducing N_2_O to N_2_. Furthermore, the elevated presence of *nirS*, *norB*, and *nosZ* genes across the three medium biofilms suggests that NO_2_^−^-N was completely denitrified to N_2_. However, the higher abundance of *narGHI* and *napAB* genes relative to *nirKS*, *norBC*, and *nosZ* in biofilms may account for the accumulation of NO_2_^−^-N in the DNBFs effluent.

Among the medium biofilms, CE2 had the highest abundance of denitrification genes, followed by CE1 and CE3 (Figure 8b). The microbial community structure positively affected the abundance of denitrification genes (standard path coefficient = 0.59, *p* < 0.05; Figure 5). The gene abundance order matched the N_RE_ observed in the corresponding DNBFs (Figure 2a). The microbial community analysis at HRT = 1 h reflects the functional maturity of biofilms under maximum hydraulic stress. While temporal shifts in microbial composition during HRT reduction remain unexplored, the high abundance of denitrifying taxa (e.g., *Proteobacteria*) and functional genes (Figure 8c) suggests that the initial colonization phase (HRT = 4 h) established a resilient community capable of sustaining activity despite operational perturbations. In conclusion, the elevated denitrification gene abundance in CE2 boosted the denitrification process, leading to its superior denitrification performance

Media with higher effective porosity and optimal porosity percentage not only increased EPS (path coefficient = 0.85, *p* < 0.01) and biofilm biomass (path coefficient = 0.82, *p* < 0.01), but also enhanced microbial community diversity and richness (path coefficient = 0.63, *p* < 0.05), improved nitrogen metabolic functions (*nirS*, *norB*, *norC*, *nosZ*), and thereby promoted denitrification performance (path coefficient = 0.95, *p* < 0.05), specifically manifested as increases in N_RE_ and R_vd_. The total effect of pore characteristics on denitrification reached 0.76 (Figure 5).

## 4. Conclusions

This study demonstrated that ceramsite media with higher effective porosity (pore size > 0.5 μm) and an optimal porosity (pore size 0.5–25 μm) percentage facilitated the accumulation of EPS and biofilm biomass. These pore structural advantages further enriched denitrifying bacteria, which in turn improved the functionality of denitrification genes, thereby enhancing denitrification performance. CE2 (effective porosity: 55.8%; optimal porosity percentage: 83.1%) exhibited superior biomass retention and EPS production, correlating with its highest denitrification activity. At the HRT = 1.5 h, CE2 achieved an R_vd_ of 0.82 kg TN/(m^3^·d), surpassing CE1 (0.74 kg TN/(m^3^·d)) and CE3 (0.68 kg TN/(m^3^·d)), and its effluent NO_3_^−^-N concentration of 7.28 mg/L met the GB5749-2022 standard. The CE2 biofilm was enriched with denitrifying bacterial taxa, such as unclassified_f_Comamonadaceae (42.7%) and unclassified_f_Enterobacteriaceae (35.3%). Functional predictions based on PICRUSt2 confirmed that a higher abundance of denitrification genes (e.g., *narG*, *nirK*, *nosZ*) in CE2, which promoted the NO_3_^−^-N removal.

The findings highlight the critical role of pore structural parameters in shaping microbial metabolic pathways and denitrification functionality, providing a mechanistic understanding for optimizing ceramsite media design. Future research could explore the applicability of these insights to other types of media, investigate long-term performance stability in large-scale water treatment systems, and integrate omics technologies to dissect the regulatory networks of key denitrification genes in pore microenvironments. From an engineering perspective, the proposed pore-structured ceramsite offers potential environmental benefits by mitigating nitrate pollution and reducing nitrous oxide emissions through enhanced terminal denitrification, as well as economic advantages via improved biomass retention and reduced operational costs. This study advances the understanding of biofilm-mediated denitrification in porous media and provides a scientific foundation for developing efficient, sustainable water treatment technologies.

## Figures and Tables

**Table 1:** Surface pore characteristics of ceramsite media.

Medium	Effective Porosity (%)	Optimal Porosity (%)	Available Porosity (%)	Percentage of Optimal Porosity (%)	Percentage of Available Porosity (%)	Fractal Dimension
CE1	47.8 ± 0.5 b	47.8 ± 0.5 a	0.0 ± 0.0 c	100 ± 0.4 a	0.0 ± 0.0 c	1.44 ± 0.06 a
CE2	55.8 ± 0.8 a	47.9 ± 1.4 a	7.9 ± 0.3 b	83.1 ± 3.6 b	16.9 ± 0.3 b	1.48 ± 0.07 a
CE3	46.7 ± 1.6 b	23.5 ± 3.2 b	23.2 ± 1.1 a	50.3 ± 6.2 c	49.7 ± 0.9 a	1.26 ± 0.03 b

Notes: The percentage of optimal porosity is optimal porosity/effective porosity; the percentage of available porosity is the ratio of available porosity to effective porosity; Values are means ± standard deviations (*n* = 3). Letters show statistically significant differences between ceramsite media treatments (*p* < 0.05).

## Data Availability

Data will be made available on request.

## Supplementary Materials

The following supporting information can be downloaded at: https://www.mdpi.com/article/10.3390/microorganisms13061187/s1, Table S1. Well water quality; Table S2. Operating parameters of DNBFs at each stage; Table S3. The reagents and experimental instruments required in the experiment; Table S4. Water quality indicators and analysis methods; Table S5. Physicochemical properties of ceramsite media; Table S6. One-way ANOVA for surface pore characteristics as affected by different ceramsite media; Table S7. Performance of DNBFs at different HRTs; Table S8. Comparative bacterial diversity analysis in biofilms of seed sludge and different pore structure media.
