# Supplementary material for: Efficiency, Microbial Communities, and Nitrogen Metabolism in Denitrification Biological Filter: Insights into Varied Pore Ceramsite Media
Source: Microorganisms. 2025 May 23;13(6):1187. doi: 10.3390/microorganisms13061187 (PMC12195522; doi:10.3390/microorganisms13061187)
Supplement: Supplementary file 1 [file microorganisms-13-01187-s001.zip › microorganisms-3490980-supplementary.pdf]

Table S1. Well water quality

Water quality indicators	pH	TDS (mg/L)	Iron (mg/L)	Manganese (mg/L)	Sodium (mg/L)	Free chlorine (mg/L)	Turbidity (NTU)	COD_{Mn} (mg/L)	Ammonia (as N) (mg/L)	Nitrate (as N) (mg/L)	Nitrite (as N) (mg/L)
Tap water	7.85	267.10	0.01	0.03	47.03	0.04	<0.50	0.66	<0.03	0.58	<0.05

Table S2. Operating parameters of DNBFs at each stage

Operational stage		Days (d)	HRT (h)	Filtering velocity (L/h)		
				CE1	CE2	CE3
Inoculation and cultivation period	\	3	4	10.0	10.0	10.0
Continuous operation period	Start-up phase	0-17	4	10.0	10.0	10.0
	Loading phase	18-41	3	20.0	20.0	20.0
		42-55	2	26.7	26.7	26.7
		56-67	1.5	40.0	40.0	40.0
		68-76	1	80.0	80.0	80.0

Note: Particle size of ceramsite medium in CE1, CE2 and CE3 reactors was uniformly 4-6mm.

Table S3. The reagents and experimental instruments required in the experiment

Index	Reagents	Experimental instruments and model	Manufacturer
COD_{Cr}	Potassium dichromate<br>Mercury sulfate<br>Sulfuric acid	Fast digestion instrument (DRB 200)	Hach (USA) Co., Ltd., United States
		UV spectrophotometer (U-T1810)	Yipu Instruments (Shanghai) Co., Ltd., China
NH_4^+-N	Nessler's reagent<br>Potassium sodium tartrate	UV spectrophotometer (U-T1810)	Yipu Instruments (Shanghai) Co., Ltd., China
NO_3^--N	Hydrochloric acid	UV spectrophotometer (U-T1810)	Yipu Instruments (Shanghai) Co., Ltd., China
NO_2^--N	Naphthalene ethylenediamine hydrochloride	UV spectrophotometer (U-T1810)	Yipu Instruments (Shanghai) Co., Ltd., China
DO		Portable DO meter (JPBJ-608)	Leici (Shanghai) Co., Ltd., China
Biomass		Electric forced air drying oven (DHG-9053A)	Yiheng Scientific Instruments (Shanghai) Co., Ltd., China
		Muffle furnace (SHZ-82A)	Zhonghuan Test Electric Furnace (Tianjin) Co., Ltd., China
		Analytical balance (CP225D)	Sartorius Trading (Shanghai) Co., Ltd., China
EPS	Anthranone<br>Sodium carbonate<br>Sodium hydroxide<br>Seignette salt<br>Copper sulfate<br>Ethyl acetate<br>Foline-phenol, Sulfuric acid	Shaking incubator (HH-1)	Zaosite Instruments (Tianjin) Co., Ltd., China
		High-speed refrigerated centrifuge (TGL-10B)	Anting Scientific Instruments (Shanghai) Co., Ltd., China
		UV spectrophotometer (U-T1810)	Yipu Instruments (Shanghai) Co., Ltd., China

Table S4. Water quality indicators and analysis methods

Water quality indicators	Methods	Instruments
NO_3^--N	Ultraviolet spectrophotometry (GB/T 5750-2006)	Ultraviolet-visible spectrophotometer (Eispectrum Instruments, U-1810)
NO_2^--N	Diazo-coupling spectrophotometric method (GB/T 5750-2006)	
NH_4^+-N	Nano reagent spectrophotometry (GB/T 5750-2006)	
COD_{cr}	Rapid Elimination Spectrophotometry (HJ/T 399-2007)	
DO	Electrochemical method	Dissolved Oxygen Measuring Instrument (REMAG JPBJ-608)
T	Direct method of measurement	

Table S5. Physicochemical properties of ceramsite media.

**Property**	**CE1**	**CE2**	**CE3**	**Test Method**
SiO_2 (%)	54.8 ± 0.3	53.1 ± 0.2	52.3 ± 0.4	XRF (GB/T 176-2017)
Al_2O_3 (%)	28.1 ± 0.2	28.9 ± 0.3	29.5 ± 0.2	XRF (GB/T 176-2017)
pH stability	7.2–7.5	7.0–7.3	6.8–7.1	Batch leaching (24 h)
Compressive strength (MPa)	8.5 ± 0.3	7.8 ± 0.2	6.2 ± 0.4	ASTM D7012

Table S6. One-way ANOVA for surface pore characteristics as affected by different ceramsite media

	**F value**	***P***	**η^2**
Effective porosity (%)	62.47	<0.001	0.954
Optimal porosity (%)	136.28	<0.001	0.978
Available porosity (%)	909.96	<0.001	0.997
Percentage of optimal porosity (%)	112.988	<0.001	0.974
Percentage of available porosity (%)	6690.88	<0.001	1.000
Fractal dimension	12.49	<0.01	0.806

Table S7. Performance of DNBFs at different HRTs

	HRT (h)	CE1	CE2	CE3	One-way ANOVA		*P* value of two-way ANOVA		
					P	η^2	Media	HRT	Medium × HRT
NO_3^--N (mg/L)	4	1.81±1.88a	1.91±1.19a	0.77±0.77a	0.370	0.153	<0.001	<0.001	<0.001
	3	2.15±1.26a	2.12±1.08a	3.40±0.75a	0.132	0.287			
	2	3.91±0.69b	3.85±0.77b	8.64±0.52a	0.000	0.934			
	1.5	11.10±1.31b	7.28±0.46c	16.12±0.76a	0.000	0.951			
	1	25.31±1.63b	22.20±0.87c	33.64±0.99a	0.000	0.912			
NO_2^--N (mg/L)	4	0.48±0.29a	0.23±0.06a	0.43±0.28a	0.262	0.200	<0.001	<0.001	<0.001
	3	1.53±0.32b	0.34±0.22c	3.38±0.35a	0.000	0.955			
	2	2.97±0.48b	2.33±0.19c	3.49±0.31a	0.001	0.701			
	1.5	3.18±0.23a	1.90±0.18b	1.83±0.36b	0.000	0.871			
	1	4.13±0.83a	3.26±0.86b	4.27±0.85b	0.170	0.256			
NH_4^+-N (mg/L)	4	0.32±0.20a	0.28±0.16a	0.26±0.07a	0.914	0.030	0.577	0.661	0.213
	3	0.43±0.16a	0.21±0.19a	0.36±0.11a	0.260	0.362			
	2	0.20±0.17a	0.36±0.08a	0.22±0.09a	0.250	0.370			
	1.5	0.32±0.07a	0.29±0.07a	0.29±0.10a	0.856	0.050			
	1	0.25±0.11a	0.15±0.10a	0.36±0.04a	0.083	0.564			
R_{vd} (kg TN/(m^3·d))	4	0.35±0.01a	0.35±0.01a	0.36±0.01a	0.643	0.071	<0.001	<0.001	<0.001
	3	0.46±0.01a	0.47±0.01a	0.43±0.01b	0.001	0.600			
	2	0.65±0.01a	0.66±0.01a	0.59±0.01c	0.000	0.938			
	1.5	0.74±0.02b	0.82±0.01a	0.68±0.02c	0.000	0.925			
	1	0.46±0.01a	0.47±0.01a	0.43±0.01b	0.000	0.927			
COD_{cr} (mg/L)	4	26.47±5.00a	25.70±3.47a	18.48±5.13a	0.141	0.479	<0.001	<0.001	0.001
	3	28.08±5.67b	27.84±8.95b	35.86±5.25a	0.288	0.340			
	2	45.83±7.14b	47.56±10.82b	68.19±7.16a	0.037	0.667			
	1.5	71.29±13.84b	59.23±9.39c	95.02±5.70a	0.025	0.706			
	1	145.96±7.16b	129.10±8.22c	164.81±4.35a	0.000	0.971			

Note: One-way ANOVA analyzed the influence of different media on water quality indicators under specific HRT. Values are means ± standard deviations (n = 5). Letters show statistically significant differences between media treatments at specific HRT ($P < 0.05$)

Table S8. Comparative bacterial diversity analysis in biofilms of seed sludge and different pore structure media

Sample name	Effective sequencing base	OTUs	Alpha					
			Sobs	Chao1	Ace	Shannon	Simpson	Coverage/%
Seed	40375±2901	261±37	261±37a	292±37a	298±37a	3.57±0.39a	0.063±0.04c	99.83±0.01b
CE1	41659±2533	112±4	112±4b	172±18b	181±29b	1.67±0.08b	0.30±0.04ab	99.87±0.01a
CE2	41746±1289	129±13	129±13b	177±30b	173±39b	1.64±0.29b	0.40±0.078a	99.88±0.03a
CE3	40003±2008	128±9	128±9b	174±33b	161±20b	1.84±0.13b	0.27±0.04b	99.88±0.03a

Note: Mean ± standard deviations (n = 3) are shown. Different lowercase letters show statistically significant differences between media treatments ($P < 0.05$).
